# The Emerging Role of MicroRNAs and Autophagy Mechanism in Pancreatic Cancer Progression: Future Therapeutic Approaches

**DOI:** 10.3390/genes13101868

**Published:** 2022-10-15

**Authors:** Evangelos Koustas, Eleni-Myrto Trifylli, Panagiotis Sarantis, Nikolaos Papadopoulos, Konstantinos Papanikolopoulos, Georgios Aloizos, Christos Damaskos, Nikolaos Garmpis, Anna Garmpi, Michalis V. Karamouzis

**Affiliations:** 1 Department of Biological Chemistry, Medical School, National and Kapodistrian University of Athens, 75, M. Asias Street, 11527 Athens, Greece; 2 First Department of Internal Medicine, 417 Army Equity Fund Hospital, 11521 Athens, Greece; 3 ‘N.S. Christeas’ Laboratory of Experimental Surgery and Surgical Research, Medical School, National and Kapodistrian University of Athens, 75, M. Asias Street, 11527 Athens, Greece; 4 Renal Transplantation Unit, ‘Laiko’ General Hospital, 11527 Athens, Greece; 5 Second Department of Propaedeutic Surgery, ‘Laiko’ General Hospital, Medical School, National and Kapodistrian University of Athens, 75, M. Asias Street, 11527 Athens, Greece; 6 First Department of Pathology, Medical School, National and Kapodistrian University of Athens, 75, M. Asias Street, 11527 Athens, Greece

**Keywords:** autophagy, chemoresistance, pancreatic cancer, microRNAs

## Abstract

Pancreatic cancer constitutes the fourth most frequent cause of death due to malignancy in the US. Despite the new therapeutic modalities, the management of pancreatic ductal adenocarcinoma (PDAC) is considered a difficult task for clinicians due to the fact that is usually diagnosed in already advanced stages and it is relatively resistant to the current chemotherapeutic agents. The molecular background analysis of pancreatic malignant tumors, which includes various epigenetic and genetic alterations, opens new horizons for the development of novel diagnostic and therapeutic strategies. The interplay between miRNAs, autophagy pathway, and pancreatic carcinogenesis is in the spotlight of the current research. There is strong evidence that miRNAs take part in carcinogenesis either as tumor inhibitors that combat the oncogene expression or as promoters (oncomiRs) by acting as oncogenes by interfering with various cell functions such as proliferation, programmed cell death, and metabolic and signaling pathways. Deregulation of the expression levels of various miRNAs is closely associated with tumor growth, progression, and dissemination, as well as low sensitivity to chemotherapeutic agents. Similarly, autophagy despite constituting a pivotal homeostatic mechanism for cell survival has a binary role in PDAC, either as an inhibitor or promoter of carcinogenesis. The emerging role of miRNAs in autophagy gets a great deal of attention as it opens new opportunities for the development of novel therapeutic strategies for the management of this aggressive and chemoresistant malignancy. In this review, we will shed light on the interplay between miRNAs and the autophagy mechanism for pancreatic cancer development and progression.

## 1. Introduction

Pancreatic cancer constitutes the fourth most frequent cause of death due to malignancy in the US, while based on the global epidemiological data, it presents an elevated incidence, which is mainly attributed to senility as well as to increased obesity rate [1]. Pancreatic ductal adenocarcinoma (PDAC)presents an early metastatic dissemination and dismal prognosis, while it is believed that it will be the most common cause of death due to malignancy in the USA by 2030 [2,3].

Some of the major risk factors that are identified are chronic pancreatitis that increases 7.2-fold the risk for pancreatic cancer [4], diabetes mellitus [5], systemic lupus erythematosus [6], lifestyle habits such as tobacco and alcohol abuse [7], obesity [8], and occupational exposure to chemical substances such as benzene, pesticides, chlorinated hydrocarbons, and asbestos [9]. Patients who present Peutz–Jeghers syndrome, which is mainly attributed to STK11 genetic mutation, have a 132-fold higher risk, while individuals with familial pancreatic is also have an increased risk for developing this malignancy up to 87 times, most commonly presenting CFTR, PRSS1, and SPINK1 mutations [10]. Another risk factor that was recently identified is periodontal disease, which is closely associated with the oral microbiome [11].Pancreatic carcinogenesis is a multifactorial event, resulting from genomic and epigenetic aberrations and deregulated signaling pathways under the influence of environmental factors. Some of the most well-documented mutant genes are *TP53*, *KRAS, SMAD4, BRCA 1/2, CDKN2A, PALB2 MSH2/6*, as well as *MLH1* and *ATM*, with *TP53, KRAS, SMAD4*, and *CDKN2A* being the most frequently reported [12].

Their management is considered a difficult therapeutic task for the majority of the cases, due to the absence of any targeted treatment. The majority of PDAC patients present to the clinician when they already have an end-stage disease, while post-operation relapse occurs in 20–25% of them. The current chemotherapeutic regimen of nab-paclitaxel–gemcitabine (FOLFIRINOX) does not significantly increase the 5-year survival (only 3%), while immunotherapeutic agents such as immune checkpoint inhibitors (ICIs) are also considered ineffective. However, they are only efficient in the few cases of PDAC that present microsatellite instability (MSI-H) [13]. Based on the aforementioned, the identification of new therapeutic targets is considered urgent [14]. MicroRNAs (miRNAs) constitute small non-coding RNA molecules of approximately 19–25 that are considered fundamental for many cell functions, such as hematopoiesis, signaling, and metabolic pathways proliferation, as well as differentiation and apoptosis [15]. They can alternate and interfere with the mRNA translation and the encoding of protein molecules, as well as with cell functions such as autophagy, proliferation, programmed cell death, and metabolic and signaling pathways. They demonstrate a binary role in carcinogenesis by the fact that they either act as tumor inhibitors or as tumor promoters. In addition, there are various studies that demonstrate the key role of microRNAs on pancreatic cancer initiation and progression, as well as their role in metastatic dissemination and chemoresistance [16]. Meanwhile, miRNAs have a key role in the autophagy pathway orchestration [17], which constitutes a multi-phased homeostatic lysosomal degradation system that is widely used by cells under conditions of stress such as lack of nutrients and hypoxia, as well as in the presence of abnormal, misfolded proteins and defected organelles [18]. A wide variety of autophagy-related genes and proteins are under the influence of miRNAs, the so-called autophagomirs. Alterations in the expression level of autophagomirs could lead to the deregulation of the autophagy pathway, a phenomenon that could lead to pathogenesis, including cancer development. Similarly, autophagy exhibits a dual role in carcinogenesis either as a tumor suppressor or promoter [17,19,20]. This characteristic creates possible druggable targets for anti-neoplastic therapy via the construction of novel agents, which can also be combined with combined with conventional chemotherapy. By this manner, autophagy regulatory points can be impeded the overall survival of the patients could be significantly enhanced [20]. Moreover, the analysis of microRNA expression profiles of pancreatic tumors could be possibly used as prognostic tools for chemoresistance and the estimation of the overall survival, while they can also be utilized as autophagy modulators [21]. In this review, we will shed light on the interplay between miRNA and autophagy in PDAC development and progression. Last but not least, we will discuss the opportunities of miRNA-based autophagy modulation for the management of this highly aggressive cancer.

## 2. An Overview of miRNA Biogenesis

**Figure 1:**
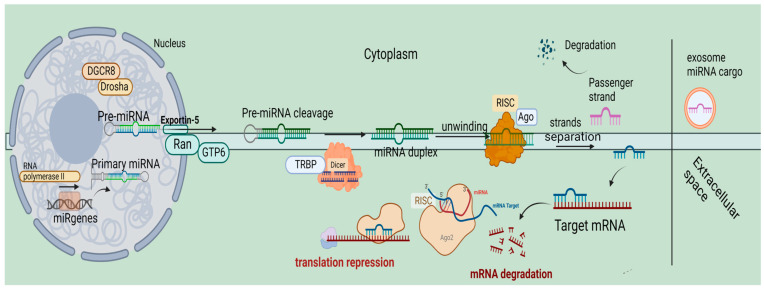
A schematic presentation of the miRNAs biogenetic mechanism. This pathway starts with the DNA transcription of microRNAs coding genes by RNA polymerase II which results in the formation of primary miRNA (pri-miRNA). The latter is cleaved by ribonuclease complex Drosha (ribonuclease III)-DiGeorge Syndrome Critical Region 8 (DGR8), resulting in the formation of premicroRNA. The pre-miRNA is transferred into the cytoplasm via Ran-GTP6 and Exportin 5 and then is cleaved by the Dicer (RNase III endonuclease)–TRBP complex. By this cleavage mature miRNA (duplex miRNA) is formed, which is double-stranded. Then, the latter is unwound with one of the strands being degraded and the other being integrated to the Argonaut protein. Then, miRNA interacts with the targeted mRNA sequence(s), which either has a repressed translation or it could be silenced or degraded. This figure was created with BioRender.com (accessed on 14 October 2022 NN24I0DSFL agreement number).

MiRNAs have a crucial role in the expression of genetic information, regulating a large number of genes (60%) [22]. The formation of miRNA is closely associated with the DNA transcription of the coding genes for microRNAs under the action of RNA polymerase II, which is an intra-nuclear procedure. The product of the above transcription is the so-called primary miRNA (pri-miRNA), which is afterward cleaved by ribonuclease complex Drosha (ribonuclease III)-DiGeorge Syndrome Critical Region 8 (DGR8), giving rise to pre-microRNA formation. The former is a quite long molecule (over 1000 bps), while the latter is shorter, including up to 100 nucleotides. The pre-miRNA is transferred into the cytoplasm via Ran-GTP6 and Exportin 5, where the Dicer (RNase III endonuclease)–TRBP complex induces its cleavage, resulting in the formation of the mature miRNA (duplex miRNA). The intracytosolic mature miRNA is composed of two strands that are being unwound, with one of the two strands being integrated with Argonaut protein (part of the RNA-induced silencing complex (RISC)), and the other, the so-called passenger strand, being degraded. The passenger strand, which cannot be loaded on the Argonaut protein, is degraded, while those which can be loaded are cleaved. The miRNA interacts with the targeted mRNA sequence(s) by being attached to the complementary mRNA sequence. The interaction between miRNA and mRNA can lead to the degradation and silencing of the latter, and it can induce the repression of its translation. All the aforementioned steps of miRNA biogenesis comprise the canonical pathway. However, there is also the non-canonical biogenesis, which is subdivided into the Dicer-independent and DGCR8/Drosha-independent pathways [23]. We demonstrate the canonical biogenetic mechanism in Figure 1.

## 3. The Emerging Role of miRNAs in Pancreatic Cancer

The role of miRNAs is considered fundamental for many cell functions such as hematopoiesis, signaling, and metabolic pathways proliferation, as well as differentiation and apoptosis [24,25,26]. However, they also take part in pathogenetic mechanisms, including cancer development, tumor invasion, proliferation, and metastatic dissemination, as well as chemoresistance [27]. Additionally, they have a significant role in mRNA translation and the encoding of protein molecules, as they closely regulate gene expression of a targeted mRNA, which is totally or partially complementary [27,28].

It has been demonstrated that miRNAs have a dual role in carcinogenesis, which is either as tumor inhibitors or as oncomiRs that promote cancer development, as a consequence of their altered expression levels in the tumors. The suppression of oncomiRs or the activation of tumor suppressor miRs are considered useful weapons against carcinogenesis [29]. There are multiple studies that demonstrate the aberrations of miRNA expression levels in pancreatic cancer, which lead to the deregulation of various cell functions and the promotion of carcinogenesis. Identification and analysis of those aberrations provide a great piece of knowledge about the nature of the pathogenesis, which could be either benign or malignant and the type of pancreatic cancer such as PDAC or pancreatic neuroendocrine tumors [30].

Futhermore, it has been shown that a wide variety of tissue-derived or circulating miRNAs are either downregulated or overregulated in PDAC. More particularly, serum from PDAC patients presents modified miRNA expression levels, which are either increased or decreased, a phenomenon that is not presented in the serum of healthy donors. Some of the circulating miRNAs that are notably increased in patients with pancreatic cancer are miR-21, miR-196a, miR-25, and miR-155, as well as miR-885-5p, miR-185, miR-2, and miR-18a. Moreover, based on several studies on miRNA profiling of PDAC bioptic specimens, some tissue-derived miRNAs that are overexpressed are miR-196a, miR21, miR-221, and miR-155. The overexpression of latter is closely associated with the initial stage of tumor progression, promoting the progression of pancreatic intraepithelial neoplasia towards high-grade lesion [31,32,33]. Meanwhile, miRNA profiling of aspiration biopsies from pancreatic malignant lesions demonstrates various downregulated or upregulated miRNAs such as miR-200c, let-7c/d/f, and miR-486-5p, miR-196a, and miR-451, respectively [30,31,32,33,34,35].

Futhermore, miRNAs levels can potentially be used as diagnostic biomarkers such as in the case of miR-135b, which constitutes a newly demonstrated diagnostic marker for PDAC and miR-25 as a marker for pancreatic malignancy identification, particularly in the early stages. In addition, another diagnostic panel that can possibly be utilized for early detection includes CA19-9, serum miR-196, and miR-16 expression levels [31,36].

Last but not least, it is necessary to underline the important interplay between miRNA and the autophagy pathway, which has a major impact on pancreatic carcinogenesis. There are approximately 100 miRNAs, the so-called autophagomiRs that take part in the orchestration of almost every step of autophagy by the targeting of autophagy-related genes and proteins. These miRNAs can be either over or downregulated during stressful cell conditions, while the same stimulus could activate the autophagy-related genes and miRNAs [37]. Later on, we will shed light on the miRNA-based autophagy regulation and its emerging role in pancreatic cancer.

## 4. An Overview of the Macroautophagy Pathway and miRNA-Based Autophagy Regulation

Autophagy is a highly regulated homeostatic catabolic pathway, which reassures the optimal conditions for cells, under stress such as deprivation of nutrients, lack of oxygen, and accumulation of potentially harmful defective intracytosolic organelles, which can be degradated and recycled [38].

**Figure 2:**
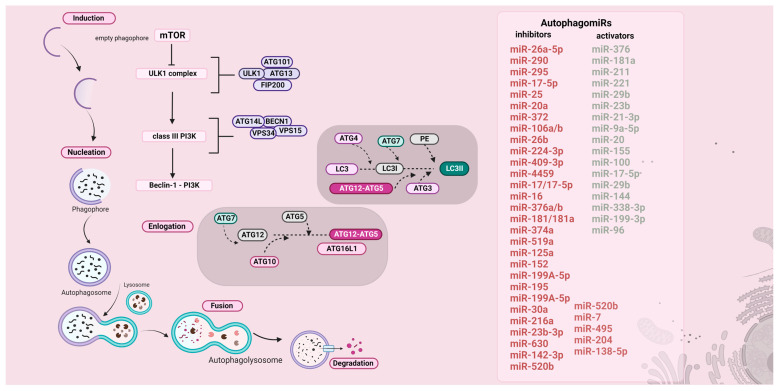
A schematic presentation of autophagy pathway and autophagomiRs. The induction of the pathway includes the inactivation of mTOR and the activation of ULK1 with the engulfment of the cargoes. Nucleation includes the activation of class III PI3K by ULK1, which is followed by the creation of the Beclin-1-PI3K complex. Afterward, the phagophore’s membrane is elongated and closed, forming the autophagosome, requiring two conjugations, which occur between LC3I-PE and ATG5-ATG12. Then follows the formation of the autophagolysosome, via the fusion of the autophagosome with the lysosome, and the (v) cargo degradation [22]. Some of the autophagomiRs that either suppress or induce autophagy pathway are demonstrated in the above scheme. This figure was created with BioRender.com (accessed on 9 October 2022) (Agreement number: AN24I5W81K). (LC3) microtubule-associated protein 1 light chain 3; (mTOR) mammalian target of rapamycin; (PE) lipid phosphatidylethanolamine; (UKL1) Unc-51-like kinase1 complex.

Shedding light on the distinct steps of this procedure, there are the following phases: (i) the induction of the pathway under stress, such as nutritional and oxygen deprivation, as well as inflammatory reactions that induce the inactivation of mammalian target of rapamycin (mTOR) and the activation of Unc-51-like kinase1 complex (ULK1), resulting in cargo engulfment. The next phase is (ii) nucleation, in which ULK1 activates (phosphorylates) the class III PI3K, a procedure that is followed by the formation ofBeclin-1-PI3K complex. The latter induces the nucleation of the phagophore [39]. Subsequently, the next step is (iii) phagophore elongation, forming the autophagosome. The development and the maturation of the autophagosome require two conjugations between ATG12 and ATG5, as well as between ATG8/microtubule-associated protein 1 light chain 3(LC3) and lipid phosphatidylethanolamine (PE). ATG12 after being activated by ATG7 forms thioester intermediates with ATG10 (E2 ubiquitin-like conjugating enzyme) and then is conjugated with ATG5. Moreover, LC3is cleaved by ATG4 and then is activated by ATG7. Afterwards, LC3I is conjugated with PE, under the participation of ATG3 and ATG12-ATG5 complex, leading to the formation of LC3II (the lipidated form of LC3I) [40,41] (Figure 1).Finally, the last two steps include the (iv) formation of the autophagolysosome via the fusion of autophagosome with lysosome and the (v) cargo degradation. All the above steps are composed of multiple structures, which could possibly act as targets for the inhibition of autophagy in the case of carcinogenesis [42]. The major steps of autophagy are presented in Figure 2.

Furthermore, miRNAs orchestrate the autophagy-related proteins that take part in the pathway in every step from induction to cargo degradation; however, autophagy might also have an auto-regulation mechanism, including the selective degradation of miRNAs [43]. Stressful stimuli such as glucose and oxygen deficiency, as well as starvation, that induce autophagy activation, also activate specific autophagomiRs, such as the family of miR30. Starting with the induction step of the autophagy pathway, there are various microRNAs that target mTOR, such as miR-7, miR-100, miR-144, miR-338-3p, miR-128, and miR-96, as well as miR-199a and miR-128. However, there are various miRNAs that activate the pathway under no stress, by suppression of the mTOR inhibition, which allows the initiation of the pathway such as miR-376 a/b, miR-181a, as well as miR-211 [44,45]. ULK1 complex is regulated by several autophagomiRs. More particularly, ULK1 is targeted by miR-26a-5p, miR-290, miR-295, miR17-5p, miR-25, miR-20a, miR372, and miR-106b, which suppress autophagy, ULK2 is targeted by miR-26b that acts as autophagy inhibitor, while FIP200 and ATG13are targeted by several suppressive miRNAs such as miR-224-3p, miR-409-3p, and miR-4459, respectively. At the level of nucleation, BECN1 is targeted by several suppressive miRNAs such asmiR-17/17-5p, miR-16, miR-376a/b, and miR-181 and by others that activate autophagy such as miR-221. Based on the stress-stimuli, BECN1 is regulated by specific miRNAs such as in the case of ionizing radiation and nutritional deprivation where it is targeted by miR-199-5p, miR-216a, and miR-376, miR-20a, as well as miR-30 family, respectively. Similarly, under the effect of chemotherapy, BECN1 levels are regulated by the miR-30 family, miR-409-3p, and miR-9 [46,47,48].

Moreover, at the level of Beclin1/VPs34, complex autophagy is targeted by several miRNAs such as miR-181a, miR-374a, miR-519a, and miR-125a which target UVRAG and suppress autophagy, miR-152, miR-199A-5p, miR-195, and miR-29b that target ATG14L, which inhibit and activate the autophagy, respectively. In addition, Beclin1 is targeted by several suppressive miRs such as miR17-5p, miR-17 and miR-16, as well as miR-376b and miR-181a. Last but not least, there are various miRs that regulate the elongation step, such as miR-23b-3p, miR-630, miR-519a, miR-224-3p, and miR-374 that inhibit autophagy, as well as miR-23b and miR-21-3p which activate it. ATG5 and ATG10 are both regulated by miR-181a, miR-630, miR-519a, and miR-374a that are autophagy suppressors, while miR-9a-5p targets the former and induces autophagy. Meanwhile, in the level of ATG12-ATG5-ATG16L1 complex, ATG16L1 is targeted by several miRs such as miR-20 and miR-142-3p that activate and suppress the pathway, respectively. Additionally, autophagy is induced by miR-20 and miR-155 that target ATG7 and ATG3, respectively. The former is also targeted by miR-520b, miR-7, and miR-106a that suppress autophagy, while miR-495 targets the latter and blocks the pathway. In the level of the LC3I-PE conjugation system, LC3II is targeted by miR-204, which suppresses autophagy. Last but not least, miR-138-5p constitutes an autophagy inhibitor, by its implication in the regulation of the SIRT1/FoxO1/Rab7 pathway, which is involved in the autophagy pathway and more particularly in the level of autophagosome maturation and fusion with the lysosome [46,49,50].In Figure 2, we demonstrate the steps of autophagy pathway, as well as the autophagomiRs that either activate or inhibit it.

## 5. The Binary Role of Autophagy in PDAC

### 5.1. Autophagy as PDAC Promoter

Autophagy has a binary role in carcinogenesis and tumor progression either as an inhibitor or stimulator. It is reported that autophagy is particularly enhanced in the cell lines of PDAC, especially in cases of premalignant pancreatic lesions, in comparison with specimens of physiological ductal tissue [51]. In PDAC cell lines, the autophagy process is highly over activated at a basal metabolic rate, in contrast with other cells, in which autophagy is stimulated under specific stimuli, such as deprivation of oxygen, nutrients, and during chemotherapy. Pancreatic malignancy is closely associated with the overregulation of autophagy, which needs to be inhibited, in comparison with multiple other types of cancers, in which autophagy suppression can possibly promote carcinogenesis [52,53].

Autophagy constitutes a homeostatic mechanism for cells that could possibly also promote the survival of cancer cells, when there is a deprivation of nutrients or oxygen in the tumor microenvironment (TME). The autophagy mechanism is often induced under hypoxia, especially in highly progressive tumors with insufficient vascularization, as a result of the action of Beclin1under the activation of hypoxia-inducible factor-1 (HIF-1a) transcription factor, which closely interacts with BH3-only protein expression, which further impedes BCL2–Beclin1 interaction [53,54]. Cancer cell redox state is also significantly associated with tumor progression and autophagy induction. More specifically, some oncogenes stimulate the expression of antioxidant Nrf2 proteins, in order to reduce the levels of reactive oxygen species (ROS), which further induces the expression of receptors for advanced glycation end products (RAGE) [55,56]. In addition, RAGE is highly increased in cases of resistant PDAC, while it is also associated with high invasiveness [57].

Autophagy is also closely interrelated with KRAS mutation, which is reported in the majority (95%) of the PDAC cases. The overexpression of the KRAS gene is significantly associated with PDAC progression via the deregulation of the autophagy pathway, while modulation of KRAS expression by anti-KRAS agents constitutes a critical strategy for PDAC management [58,59,60]. More particularly, it is demonstrated that mutation of KRAS in PDAC is critical for the progression of PDAC via the upregulation of autophagy. However, the acute inhibition of the above leads to an additional increase in the autophagic flux [61]. Similarly, the ERK1/2 pathway is also closely associated with KRAS mutant cell lines of PDAC, in which autophagic flux, as well as LC3II levels, are further increased when suppression of ERK is applied [62,63,64].

Under the condition of oxidative stress, autophagy response is elicited by High Mobility Group Box 1 (HMGB1), which has multiple key functions. HMGB1 overexpression is closely associated with carcinogenesis as it modulates the turnover of LC3by controlling LC3 ubiquitination-like reactions, while it also modulates p62 and the formation of autophagolysosome. Additionally, HMGB1 also interacts with Beclin1via its regulatory effect on Bcl2 that leads to the release of Beclin1 and the induction of an indirect stimulatory effect on RAGE. The result of the aforementioned phenomenon is the induction of the autophagic pathway and the enhancement of chemoresistance [65]. Moreover, endoplasmic reticulum stress constitutes another factor that can potentially induce autophagy in PDAC under stress, such as nutrient and oxygen deprivation, as well as the presence of unfolded or misfolded proteins. ER stress induces the unfolded protein response (UPR), which is a signaling pathway that aims for either the ER homeostasis re-establishment or cell apoptosis, in case the restoration is not achieved [66].

### 5.2. Autophagy as a Tumor Suppressor in Pancreatic Cancer

Autophagy also serves the role of tumor suppressor in the early stage of disease, limiting tumor growth and development, as well as enhancing survival. During the early stage of malignancy, the autophagy mechanism is considered protective for the cells against several injurious stimuli, such as ROS. By this manner, ROS do not interrupt the process of LC3 delipidation by altering the active site, resulting in the aggregation of the lipidated form of LC3 and the enhancement of autophagosome formation. The above phenomenon is implied by the fact that mice who lack autophagy mechanisms and present KRAS mutation have an increased risk of developing PanIN, which constitutes a precursor of PDAC [67]. Last but not least, the autophagy pathway also presents the role metastasis suppressor by inhibiting tissue transglutaminase (TG2) which is implicated in metastatic dissemination [68].

## 6. The Binary Role of miRNAs in PDAC

### 6.1. MiRNAs as Tumor Suppressors in PDAC

It has been demonstrated that several miRNAs act as tumor suppressors in pancreatic cancer, either by targeting the autophagy pathway or by regulating several pivotal pathways for cell function [69].It has been demonstrated that the levels of miR-451a are elevated in PDAC, exhibiting a tumor effective role via regulating the expression of several important genes, such as Activating Transcription Factor 2 (*ATF2*), what is a housekeeping gene, and *RAB14* (RAS Oncogene Family) [70]. Other tumor suppressive miRNAs that have been identified are miR-30c, miR-340, miR-506, miR-143-3p, miR-203a-3p, miR-519d-3p, miR-375, miR-216b, miR-142, miR-455, and miR-1181. Similarly, several other miRNAs that have been demonstrated includingmiR-15a, miR-1179, miR-135a and miR-183, miR-365a-3p, miR-300, and miR-202. More particularly, miR-30c targets TWF1 and induces arrest of G1-phase of cell cycle and apoptosis, implying its role as a tumor0suppressive miRNA, while in cases where miR-30c was decreased, re-expression led to favorable anti-neoplastic effects [71]. MiR-340 overexpression is closely associated with the regulation of Bicaudal-D2 (BICD2), leading to the inhibition of pancreatic malignant cell growth and progression, implying the anti-neoplastic potential of miR-340/BICD2 axis [72].MiRNA-506 constitutes another miRNA that acts as tumor suppressor and significantly reduces the PDAC progression, although this is achieved when it is overexpressed [73].

Furthermore, miR-143-3p, which targets KRAS, is usually downregulated in pancreatic cancer. However, enhancement of miR-143-3p levels leads to downregulation of the MERK/ERK signaling pathway and prevents the cancerous transformation of the pancreatic cells [74]. MiR-203a-3p constitutes another miRNA that is closely associated with the regulation of fibroblast growth factor 2 (FGF2) which promotes pancreatic cell proliferation and invasiveness, while its overregulation showed favorable anti-cancer effects by suppressing the epithelial–mesenchymal transition (EMT) [75]. Additionally, when miR-519d-3p is overexpressed, it is considered another tumor suppressor for PDAC by regulating Wnt signaling pathway, through targeting ribosomal protein S15A (RPS15A). Although the level of miR-519-3d is usually reduced in pancreatic malignant tissue and the levels of RPS15A are increased, enhancement of miR-519d-3p leads to a significant decrease of RPS15A expression and suppression of pancreatic cell growth [76]. Furthermore, miR-375 is found downregulated in PDAC tissue, a phenomenon that is mainly associated with metastatic and lymphatic dissemination. However, overregulation of this miRNA induces apoptosis of the pancreatic cancer cells, implying its tumor-suppressive effect [77]. Similarly, miR-216b is found with low expression in PDAC samples, with a concomitant increase of KRAS levels; however, its overregulation induces KRAS suppression. Additionally, overregulation of miR-216b targets the translationally controlled 1(TPT1) tumor proteins, leading to tumor growth suppression [78]. Moreover, another miRNA that is identified as tumor suppressor when overexpressed is miR-142, which targets HIF-1a and limits the tumor growth and invasiveness. However, the aforementioned miRNA is usually found downregulated in pancreatic malignant tissue samples [79].

In addition, it has been demonstrated that miR-455-3p suppresses EMT and TAZ expression and induces cell apoptosis [80]. Meanwhile, miR-1181 suppresses the proliferative and invasive behavior of pancreatic cancer cells by inhibiting the expression of signal transducer and activator of transcription 3 (STAT3) [81]. It has to be noted that miR-15aexpression induces cell cycle arrest, suppresses PDAC cell proliferation, and increases chemosensitivity to gemcitabine [82], while when the levels of miR-1179 are enhanced, the suppression of E2F transcription factor 5 is possible, which leads to pancreatic cell growth inhibition [83]. Moreover, miR-135a, by targeting Bmi1, suppresses PDAC growth [84], whereas miR-183 downregulation increases sensitivity to chemotherapeutic agents, including gemcitabine and 5-fluorouracil, and limits pancreatic cell growth [85].

Likewise, miR-365a-3psuppresses NF-Kb, which is correlated with PDAC invasiveness [86], miR-300 inhibits EMT and cancer cell growth by targeting Cullin 4B (CUL4B) [87], andmiR-202 upregulation suppresses proliferation by interfering with glycolysis [88]. Last but not least, based on the study of Zhang et al., miR-326 overexpression demonstrated anti-proliferative effects on PDAC, while its inhibition elicited an increased tumor proliferation and progression [89].

### 6.2. MiRNAs as Tumor Promoters in PDAC

There are multiple reports that demonstrate the involvement of miRNAs in different stages of carcinogenesis, including the development of chemoresistance and metastatic dissemination. MiR-186 is commonly overexpressed in PDAC, while targeting Nuclear Receptor Subfamily 5 Group A Member 2 (NR5A2) significantly influences tumor cell proliferation and dissemination, which are notably promoted [89]. Tumor initiation in PDAC is closely associated with the reduced levels of miR-34; however, when miR-34 is restored, it leads to the suppression of cancer stem cells (CSCs) [90]. Similarly, as it was previously referred to, several downregulated miRNAs lead to pancreatic cancer cell proliferation and migration, such as miR-30c, miR-506, miR-143, miR-203-3p, miR-519d, miR-375, as well as miR-216b, miR-142, and miR-1179. However, the re-expression of these miRNAs constitutes a weapon against PDAC proliferation, migration, and metastasis [69].

Moreover, miR-21 is considered a major oncomiR that targets tumor-suppressor genes and induces the reduction of apoptotic mechanism. MiR-21expression is closely associated with the regulation of the epidermal growth factor (EGF) signaling pathway, while it induces EGF-related pancreatic cancer cell proliferation, deregulates the cell cycle function, and suppresses apoptosis. Meanwhile, miR-21, by targeting several other signaling pathways such as PI3K/AKT and Ras-Raf-MEK-ERK pathways, induces PDAC cell proliferation [91].

Furthermore, several other miRNAs have been identified in PDAC samples, promoting oncogenesis. Some of the key oncogenic miRNAs include miR-196b, miR-221, miR-18a, miR-212, miR-301a-3p, miR-205, miR-29a, and miR-17-5p. Similarly, oncomiRs are also considered the miR-191, miR-182, miR-374a, miR-10band miR-1469-5p. More particularly, miR-196b targets cell adhesion molecule 1(CADM1) and constitutes the chief regulator of proliferation and late apoptosis [92].

MiR-506 constitutes another oncogenic miRNA; however, as it was previously referred to, it can act as a suppressor of tumor growth [93]. Another oncomiR is miR-221, which is usually found overregulated in PDAC and leads to apoptosis suppression and increased cancer cell proliferation and metastatic dissemination [94].Moreover, the miR-18a level is found increased in the circulation, as well as in PDAC tissue specimens [95]. Similarly, miR-221 levels were also found to increase in PDAC patients, while miR-301a-3p targets SMAD4, which is closely associated with the invasiveness and migratory behavior of the tumor [96,97].

Additionally, miR-205 targets the tumor suppressor gene Adenomatous polyposis coli (APC) and is closely associated with the proliferation of the cancer cells via its effect on the Wnt/β-catenin signaling pathway [98]. MiR-29a is also considered an oncogenic miRNA, which is related to the migratory and invasive behavior of PDAC, while miR-17-5p (part of the miR-17-92 cluster) interferes with the cell cycle and promotes the proliferation of pancreatic cancer cells by disrupting retinoblastoma-like protein 2 (RBL2)/E2F Transcription Factor 4 (E2F4)-repressing complexes [99,100].

Moreover, miR-191 is closely associated with TME via its effect on extracellular matrix modification, promoting the metastatic dissemination of pancreatic cancer cells [101]. MiR-182 is another oncogenic miRNA that promotes the proliferation of cancer cells by interfering with the β-catenin pathway. More particularly, the levels of miR-182 are increased in the pancreatic malignant tissue, promoting PDAC progression and growth by targeting β-transducin repeat-containing protein (β-TrCP2) [102].

Meanwhile, miR-374a targets Secernin 1 (SRCIN1) and reduces its levels, leading to the migratory and proliferative behavior of pancreatic cancer cells, as well as to EMT [103]. MiR-10 is another miRNA that is overregulated in PDAC, while lower levels of miR-10 are associated with better overall survival, a favorable response to neoadjuvant or surgical therapeutic strategy, as well as with an elongated interval without metastasis [104]. Last but not least, upregulation of miR-1469-5p is closely associated with cancer cell proliferation and the migratory behavior of PDAC by targeting and regulating the N-Myc Downstream Regulated 1 (NDRG1)/NF-κB/E-cadherin axis [105]. In Table 1, we present a summary of miRNAs that act as tumor promoters or suppressors in PDAC.

## 7. The Interplay of Autophagy and miRNAs in PDAC

MicroRNAs have an important regulatory role for autophagy. Some miRNAs promote the autophagy pathway and lead to pancreatic cancer cell destruction, whereas others enhance the anticancer effect, via the inhibition of autophagy [106].

### 7.1. MiRNA-Induced Autophagy Inhibition as a Tumor Suppressor

Starting with the induction step, the mTOR is regulated through miR-129−3p (nuclear factor erythroid two like-2 (Nrf2)/miR-129-3p/mTOR Axis). Nrf-2 is closely associated with the overregulation of miR-129-3p and leads to the inhibition of mTOR and the subsequent induction of the autophagy pathway. This phenomenon leads to increased resistance to high-dose cytarabine (HDACi), while it can be suppressed by the downregulation of the Nrf2-miR-129-3p axis [107]. MiR23b can potentially limit autophagy and increase the therapeutic effect of radiotherapy on PDAC cells bytargetingATG12. PDAC is characterized by an increased autophagy activity, which is further enhanced by chemo-radiotherapy, resulting in resistance to both treatment modalities. It is reported that enhancement of miR-23b expression might be advantageous for patients prior to radiotherapy, which constitutes the major therapeutic strategy for this malignancy. Increased levels of miR-23b are closely associated with radiosensitivity, while miR-23b expression screening is considered a necessity for the augmentation of the anti-cancer effect of chemoradiotherapy [108]. Moreover, other miRNAs that enhance the chemosensitivity of PDAC cells to gemcitabine and decrease the autophagy pathway are miR29a and miR-29c. The latter inhibits autophagy and increases sensitivity to GEM via the downregulation of autophagy-related protein expression, while it subsequently blocks the fusion of lysosome-autophagosome. This phenomenon is achieved by the inhibition of protein expression for ATG9A and TFEB, which is crucial for the fusion [109,110,111]. Blockage of autophagy is also reported after the application of miR-590-5p (that targetsATG3) in vitro, which is a crucial component of the LC3I-PE conjugation system [112]. Last but not least, chemosensitivity is also promoted by the application of miR-410-3p, which targets HMGB1 and leads to the increased autophagic flux under oxidative stress [113].

Additionally, autophagy and Wnt/β-catenin pathways are suppressed by mir-619-5p, via the downregulation of ATG14and Pygo2, respectively. Long noncoding RNA (lncRNA) is also implicated in PDAC, especially for the lncRNA plasmacytoma variant translocation 1 (PVT1), which is closely associated with tumor progression and chemoresistance to gemcitabine in pancreatic malignancy. PVTI is characterized as a “sponge” for miRNAs in order to suppress their activities [114,115]. PVT1 acts as a sponge for miR-619-5p and induces the overregulation of autophagy via ATG14 regulation (PVT1/miR-619-5p axis) and the Wnt/β-catenin pathway by Pygopus2 (Pygo2) overexpression. This phenomenon leads to the deregulation of miRNA function with several effects on the genome. Modulation of PVT1 that takes part in pancreatic carcinogenesis and chemoresistance to gemcitabine, is also considered a potent druggable target, while it is proven that the knockdown of PVT1could suppress the autophagy mechanism and limit the chemoresistance to gemcitabine by miR-143/HIF-1α/VMP1 axis [114,115,116,117,118,119,120]. Another example of miRNA-related autophagy inhibition that suppresses PDAC grow this mediated by miR-7 and miR-372 [121,122]. The former induces upregulation of mTOR, inhibits autophagy induction, and suppresses tumor growth, proliferation, and metastatic dissemination. The latter is closely associated with ULK1 regulation via autophagy suppression, which is significantly involved in the inhibition of cell proliferation. Last but not least, miR-376a is another miRNA that suppresses the autophagy pathway, by targeting Beclin1 and ATG4C [121,122,123].

### 7.2. MiRNA-Induced Autophagy Activation as Tumor Suppressor

Some autophagomiRs promote the autophagy pathway and lead to pancreatic cancer cell destruction such as miR-506 and miR-221. It is reported that the replacement of miR-506-3p in PDAC has various effects on apoptosis, autophagy pathway, and on mitochondrial modifications not only in vitro but also in vivo. Based on the study of various pancreatic cancer entities, miRNA-506-3p was found downregulated in the majority of them (71%), whereas PIM-3 proto-oncogene was found overregulated [73]. Based on the above data, the downregulation of miR-506 expression was closely related to pancreatic tumor progression, with the less differentiated pancreatic tumors having a lower expression of miR-506, in comparison with moderate and well-differentiated tumors. A decrease in cell proliferation (almost 80%) was observed after transient miR-506 transfection, regardless of the initial expression of miR-506, in comparison with the negative control. Furthermore, transfection of PaTu-8988t cells that express a mimic of miR506-3p resulted in the enhancement of apoptosis (three times higher). Meanwhile, the levels of reactive oxygen species (ROS), which are related to the mitochondria-induced apoptotic mechanism, were increased in the post-transfection period of 72h. The autophagy pathway was also modified after the transfection of PaTu-8988t, with a 2–3 times higher level of LC3-II/LC3I ratio, implying the increased activation (40%) of autophagy machinery in the transfected cells [73].

In addition, it has to be underlined that miR-506 also activates autophagy by interacting with STAT2-BCL2-BECN1 pathway. Moreover, miR-221 induces autophagy and apoptotic mechanisms and facilitates the limitation of pancreatic cancer cell proliferation. More particularly, miR-221 is closely related with the suppression of deacetylase histone deacetylase 6 (HDAC6), which is involved in the clearance of protein aggregates and with the autophagy pathway, while decreased miR-221has an oncogenic potential, via the overexpression of HDAC6 [124].

### 7.3. MiRNA-Induced Autophagy Modulation in PDAC Progression

Last but not least, there are several reports of miRNA-based autophagy pathway modulation promoting PDAC progression. An example ismiR-23b (targets ATG12) which enhances the autophagy pathway and increases radioresistance. More particularly, it is observed that radioresistant pancreatic tumors exhibit downregulation of miR-23b and overregulate autophagy pathway, in comparison with non-radioresistant tumors. Additionally, re-expression of miR-23bresensitizes the pancreatic tumor cells to radiotherapy and suppresses autophagy (radiotherapy-related) by targeting ATG12 [125]. Moreover, many cancers, including PDAC, express NF-E1 (also called Ying-Yang 1) transcription factor. The NF-E1, which is involved in many malignancies, including PDAC, targets miR-30a, which regulates Beclin1, and ATG5. More particularly, YY1 pro-autophagic effects are regulated via the circuit of YY1/MiR-30a [126,127].

## 8. Future Therapeutic Strategies Based on miRNA-Related Autophagy Modulation

Novel therapeutic strategies have been revealed by taking advantage of the various targets of miRNAs on the autophagy pathway and its binary role in pancreatic carcinogenesis. MiRNA utilization can enhance the therapeutic effects on PDAC by suppressing or promoting the autophagy pathway. Based on the aforementioned, some of the miRNAs that target autophagy and suppress pancreatic cancer cell proliferation are miR-7, miR-221, and miR-506, which demonstrate a great therapeutic potential for PDAC management by enhancing autophagy. Meanwhile, there are studies about nanoparticles that are embedded within miR-212, which enhance the sensitivity of PDAC cells to doxorubicin [128]. Moreover, upregulation of miR-23b levels in radioresistant PDAC leads to sensitization of tumor cells to radiotherapy [126], while enhancement of miR-29c regulates USP22 increases the sensitivity of pancreatic cancer cells to chemotherapy [129]. However, further research on miRNA profiling of pancreatic tumors and a better understanding of their effect on the autophagy pathway are considered crucial for the management of PDAC.

## 9. Conclusions

Pancreatic cancer constitutes a highly aggressive malignancy, despite the novel therapeutic modalities. This is mainly attributed to the late diagnostic time and the significant chemoresistance of pancreatic cancer. The analysis of PDAC molecular background, including miRNA profilingas well as understanding of miRNA and autophagy binary role in pancreatic carcinogenesis could open up new therapeutic opportunities. Deregulation of miRNA expression levels has been closely related to cancer cell growth and progression, while it is also implicated in metastatic dissemination and chemoresistance. Based on the aforementioned, the manipulation of miRNAs that modulate metabolic and signaling pathways as well as programmed cell death and autophagy is considered a powerful weapon against PDAC. However, shedding light on miRNA PDAC profile, as well as on the significant interplay between miRNA levels and autophagy pathway is considered pivotal for the discovery of novel therapeutics trategies.

## Figures and Tables

**Table 1:** A summary of miRNAs that act as tumor promoters or suppressors in PDAC.

Tumor Suppressor MiRNAs	Tumor Promoters (OncomiRs)
miR-143-3p [74]	miR-301a-3p [97]
miR-203a-3p [75]	miR-1469-5p [105]
miR-519d-3p [76]	miR-17-5p [100]
miR-365a-3p [86]	miR-186 [89]
miR-451a [70]	miR-34 [90]
miR-30c [71]	miR-196b [92]
miR-340 [72]	miR-506 [93]
miR-506 [73]	miR-221 [96]
miR-375 [77]	miR-18a [95]
miR-216b [78]	miR-21 [91]
miR-142 [79]	miR-205 [98]
miR-455-3p [80]	miR-29a [99]
miR-1181 [81]	miR-191 [101]
miR-15a [82]	miR-182 [102]
miR-1179 [83]	miR-374a [103]
miR-135a [84]	miR-10b [104]
miR-183 [85]	
miR-300 [87]	
miR-202 [88]	
miR-326 [89]	

## Data Availability

Not applicable.

## Abbreviations

activating transcription factor 2	ATF2
adenomatous polyposis coli	APC
bicaudal-D2	BICD2
cullin 4B	CUL4B
DiGeorge syndrome critical region 8	DGR8
E2F transcription factor 4	E2F4
epithelial–mesenchymal transition	EMT
fibroblast growth factor 2	FGF2
high mobility group box 1	HMGB1
microRNAs	miRNAs
N-Myc downstream regulated 1	NDRG1
pygopus2	Pygo2
RNA-induced silencing complex	RISC
ribosomal protein S15A	RPS15A
secernin 1	SRCIN1
unc-51-like kinase1 complex	ULK1
cancer stem cells	CSCs
cell adhesion molecule 1	CADM1
chaperon-mediated autophagy	CMA
epidermal growth factor	EGF
high-dose cytarabine	HDACi
hypoxia-inducible factor-1	HIF-1a
lncRNA plasmacytoma variant translocation 1	PVT1
long noncoding RNA	lncRNA
lysosomal membrane protein 2A	LAMP-2A
mammalian target of rapamycin	mTOR
messenger RNA	mRNA
microsatellite instability	MSI-H
nuclear factor erythroid two like-2	Nrf2
pancreatic adenocarcinoma	PADC
phagophore assembly site	PAS
phosphatidylethanolamine	PE
phosphatidylinositol-3-phosphate	PtdIns3P
primary miRNA	pri-miRNA
reactive oxygen species	ROS
receptors for advanced glycation end products	RAGE
retinoblastoma-like protein 2	RBL2
ribonuclease complex Drosha	ribonuclease III
signal transducer and activator of transcription 3	STAT3
tumor microenvironment	TME
tumor protein, translationally controlled 1	TPT1
unfolded protein response	UPR
β-transducin repeat-containing protein	β-TrCP2
